# Linking paranormal and conspiracy beliefs to illusory pattern perception through signal detection theory

**DOI:** 10.1038/s41598-023-36230-0

**Published:** 2023-06-16

**Authors:** Petra Müller, Matthias Hartmann

**Affiliations:** 1 Faculty of Psychology, UniDistance Suisse, 3900 Brig, Switzerland; 2 Institute of Psychology, Universität Bern, 3012 Bern, Switzerland

**Keywords:** Psychology, Human behaviour

## Abstract

Previous research indicates that irrational beliefs (Paranormal beliefs & conspiracy theory endorsement) are associated with the perception of patterns in noise, but the previous findings do not conclusively describe this relationship. This study aims to disentangle the underlying parameters of this association by applying a signal detection theory approach, thus allowing to distinguish illusory pattern perception (false alarms) from perceptual sensitivity and response tendencies—while also taking base rate information into account. Results from a large sample (N = 723) indicate that paranormal beliefs relate to a more liberal response bias and a lower perceptual sensitivity, and that this relationship is driven by illusory pattern perception. Such a clear pattern could not be observed for conspiracy beliefs, for which the increase in false alarm rates was moderated by the base rate. The associations between irrational beliefs and illusory pattern perception were however less substantial compared to other sources of variance. Implications are discussed.

## Introduction

Irrational and unwarranted beliefs are widespread in human societies^1,2^ and have been a topic of scientific inquiry for decades. Of particular interest are paranormal beliefs and conspiracy theory endorsement, which are characterized by the acceptance of unproven or unfalsifiable claims that challenge mainstream knowledge and scientific evidence. This area of study has gained increasing attention, as the prevalence of such beliefs—especially beliefs in conspiracy theories—in modern society has become a cause for concern, for example in the context of the global 2020 COVID-19 pandemic^3,4^. Conspiracy theories are commonly defined as the belief that important events are caused by secret plots of powerful evil individuals or groups^5^ and may have serious consequences, not just for public health^6^, but also for social coexistence^2^ and political participation^7^. Believers in the paranormal believe in phenomena that violate the scientifically founded principles of nature, such as beliefs in ghosts, magic, supernatural powers, entities or energies^8^ and paranormal beliefs as well may not always be benign^9^. Although seemingly disparate, conspiracy and paranormal beliefs are suspected to have common underlying mechanisms as they are frequently found to be correlated^10–12^.

Studies have suggested that a more intuitive and less analytical thinking style is associated with both conspiracy theory endorsement (for an overview, see Binnendyk and Pennycook^13^) as well as paranormal beliefs^14,15^. Another mechanism that seemingly underlies both paranormal beliefs and belief in conspiracy theories manifests in a misperception of chance and randomness, that is a heightened tendency to perceive random stimuli as non-random, for example by attributing agency where it does not exist (conspiracy beliefs^16^; paranormal beliefs^17^) or being susceptible to the *Conjunction Fallacy* (conspiracy beliefs^18,19^; paranormal beliefs^20^). Illusory pattern perception, the tendency to perceive meaningful patterns or connections in random or ambiguous stimuli, is also suspected to be an underlying factor linked to irrational beliefs^21^. Illusory pattern perception is assumed to be a side effect of the human inclination to perceive patterns to make sense of the world^22,23^. The relationship between paranormal beliefs in particular and the illusory pattern perception in random sequences has been demonstrated frequently^24–28^ and can also be observed in visual pattern detection tasks such as face or object detection^28–31^ and image categorization^32^. The link between illusory pattern perception and conspiracy theory endorsement has been tested less extensively, but findings suggest they might be connected in a similar fashion^28,29^, although conflicting findings exist^19^.

As pointed out by van Prooijen et al.^28^ however, it has not yet been conclusively explained whether irrational beliefs (paranormal beliefs and conspiracy theory endorsement) are associated with a heightened pattern sensitivity, a tendency to produce false alarms, or if a response bias such as a general yes-say tendency is at the core of these differences. In order to differentiate between these alternatives, a task is required that allows us to quantify hits, misses, correct rejections and false alarms. Signal detection theory approaches allow to differentiate between perceptual sensitivity and response bias for both the perception of noise and real patterns.

To the best of our knowledge, only four studies so far have addressed this question with a signal detection theory paradigm, and the limited evidence suggest that believers have a more liberal response criterion, favouring false alarms, compared to sceptics^17,30,31^ and differences in perceptual sensitivity were observed^17,30,33^. With the exception of the study conducted by Seymour et al.^33^, the aforementioned studies focused on extreme group comparisons, and at that particularly on beliefs in the paranormal, not other forms of irrational beliefs. The sample sizes in all these studies were well below the recommended sample size of N = 250 for correlational studies^34^. Some of the studies also lack “true” signal-stimuli, or can not assess the specific role of false alarms for sensitivity or response criterion^31,33^. Furthermore, response bias in signal detection tasks can be affected by participants response strategy and their knowledge about the task, such as the base rate information^35,36^—an aspect that hasn’t yet been examined in the context of irrational beliefs, yet could be influential as *Base Rate Neglect* has been linked to paranormal beliefs^37^.

With our present study we examined the relationship between irrational beliefs, namely the belief in conspiracy theories (both a general conspiracy mentality and specific conspiracy beliefs) and paranormal beliefs with illusory pattern perception in a large scale experiment (N = 723), applying a signal detection theory paradigm. Two sets of stimuli were used, one containing face and the other containing house targets, to additionally address the question whether the human proneness to face perception^38^ influences the relationship between irrational beliefs and illusory pattern perception. All participants completed one block of trials with 50% signal stimuli, followed by a second block wherein the percentage of signal stimuli was either higher (75%, *high base rate condition*) or lower (25%, *low base rate condition*).

We expect the different forms of irrational beliefs (paranormal beliefs, conspiracy mentality, COVID-19 conspiracy beliefs) to be correlated^12,39^. Based on the limited evidence from previous studies, we assume that higher levels of paranormal beliefs are associated with a more liberal response criterion and a higher false alarm rate^30,31^, and we will examine for the first time if such a relationship can also be observed for conspiracy beliefs. We further expect the response criterion to shift towards a more liberal response criterion in the high base rate condition and towards a more conservative criterion in the low base rate condition, and we will explore if this shift is less pronounced the higher the level of irrational belief endorsement, and more generally, whether base rate or the type of stimuli (face vs. house) moderates the relationship between irrational beliefs and pattern perception.

## Method

### Participants

Seven hundred and forty-three participants completed the study, out of which 18 were identified to have a disproportionately long or irregularly short (less than 20 min) experiment duration and were subsequently excluded. Duration was considered to be disproportionately long if it was identified to be an outlier applying Rosner outlier test (rosnerTest function from the EnvStats R package^40^). We deemed an experiment duration of at least 20 min to be sufficient for a serious attempt. Another two participants were excluded due to disproportionately poor task performance: One participant responded “signal” in 99 out of 100 trials in experiment Block 2, the other was identified as an outlier by Rosner outlier test with a sensitivity index $d^{\prime }$ = $-0.033$ in experiment Block 2. It can be expected that paranormal and conspiracy beliefs only explain a small proportion of variance in a perceptual decision task. Thus, applying Cohen’s convention^41^, we assumed a small effect size (r = .10). Accordingly, we would need a minimum sample size of *n* = 614 to reliably detect (with probability greater than 0.8) a small effect size of $\ge 0.1$, assuming a one-sided criterion for detection that allows for a maximum Type I error rate of a = .05. The final sample consisted of 723 participants aged between 17 and 78 years (*M* = 30.4, *SD* = 14.0), of which 383 (53.0%) identified as female, 330 (45.6%) as male and 10 as other (1.4%). The study was conducted in the context of an educational course of the psychology curriculum of the University of Bern. Consequently, 43 undergraduate psychology students acted as experimenters and recruited participants from their personal circle of acquaintances and relatives. Given that the number of participants that each experimenter was required to recruit was predetermined by the educational course, our sample size was larger than the a-priori defined minimum sample size. We confirm that no analyses were performed before the final sample size was reached. Data collection took place in autumn 2021. Thirty-three participants (4.6%) indicated elementary school, 198 (27.4%) apprenticeship/vocational school, 308 (42.6%) graduate school, and 184 (25.4%) university graduation as their highest educational qualification. All participants gave their written informed consent prior to the study. The study protocol was approved by the Ethics Committee of the Human Sciences Faculty of the University of Bern and the study was performed in accordance with all ethics ordinance guidelines stated by the Ethics Commission of the Human Sciences Faculty of the University of Bern.

### Stimuli

**Figure 1:**
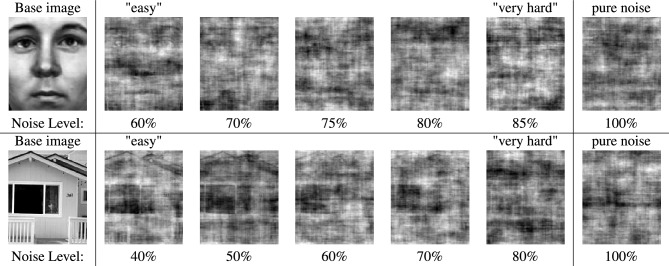
Example stimuli of faces (upper row) and houses (lower row) used in the experiment with their corresponding base image and noise levels (amount of scrambled phase matrix values) from left to right.

A set of 50 grey-scale base images (25 faces, 25 houses, size: 131 by 156 pixels) was used to generate the stimuli. Base face images were selected from face database, MPI for Biological Cybernetics, Germany^42^), base house images were adopted from van Elk^32^ and supplemented by similar images from a data set consisting of exterior images of houses^43^. Fast Fourier Transforms (FFT) of these base images were computed to obtain 50 magnitude and 50 phase matrices, then stimulus images were produced by applying the inverse FFT (IFFT) of the average magnitude matrix and individual phase matrices with varying amounts of added noise by scrambling a percentage of randomly selected values^42^ for each image category. For the signal trials, we targeted six levels of noise to have a reasonable range of difficulty. The appropriate percentages of added noise for face and house stimuli were determined based on the results of a pretest (n = 38): The results indicated that for faces, stimuli with up to 70% of scrambled phase matrix values were correctly identified as “contains a face” with an accuracy greater than 80% by participants. For houses however accuracy started to fall below 80% at 60% scrambled phase matrix values. Based on this observation, it was decided to apply a range of 60–85% (60%, 70%, 75%, 80%, 85%) of scrambled phase matrix values for face images and 40–80% (40%, 50%, 60%, 70%, 80%) for house images to obtain a similar stimulus difficulty for both image categories. To create 100% noise images, 100% of phase matrix values were scrambled before calculating the IFFT. By this method, a total of 125 noisy faces, 125 noisy houses and 125 100% noise images were generated. Examples are shown in Fig. 1.

### Irrational belief assessments

Paranormal beliefs were measured with the Proneness to the Paranormal Scale^39^ which consists of 12 items that were rated on a 7 point scale ranging from 1 (*I do not agree at all*) to 7 (*I fully agree*). Items include statements such as “Some people have an extrasensory ability to read the thoughts of others or transfer them to others”. or “Some decisions or events in our lives are influenced by experiences we have had in a previous life”. Conspiracy Mentality was measured with the 7 item “Conspiracy theory ideation” sub-scale of the Conspiracy Mentality Scale^44^. Example items include “Events on the news may not have actually happened”. and “The government or covert organizations are responsible for events that are unusual or unexplained”. These items were rated on a 7 point scale (1 = *I do not agree at all*, 7 = *I fully agree*). To assess COVID-19 specific conspiracy theory beliefs, 7 items were partially adapted from Hartmann and Müller^39^. These items were rated on an 11 point scale ranging from 1 (*certainly not*) to 11 (*certainly*) and included statements such as “Pharma companies use the SARS-CoV-2 pandemic to test novel vaccines and medication on a large number of people.” or “The government uses the SARS-CoV-2 pandemic to justify and intensify the surveillance of citizens.”. Questionnaire items were presented in random order and were intermixed with items from other scales unrelated to the study at hand (see Table S7 in the Supplemental Materials associated with this article). Scale metrics are reported in Table 1. The full list of items of the three scales is provided in the Supplemental Materials associated with this article, Tables S2, S3 and S4 respectively.

### Procedure

The experiment was designed in the lab.js Builder framework^45^ and deployed on an Apache web server (2.4.25) running on a Debian operating system for the purpose of data collection. Data collection took place during the last quarter of 2021. Experimenters used their own laptops for data collection and accessed the experiment via a link. It took participants between 20 and 76 min to complete the experiment (*M* = 38.3 min, *SD* = 9.7 min). Half of the participants were assigned to the ‘faces’ condition, while the other half completed the ‘houses’ version of the experiment. Participants were instructed to view images in succession and to decide for each image whether it contains the stimulus (e.g. a house or a face) or not. During each trial, after a fixation screen (500ms), an image was presented for up to 3500 ms during which participants decided whether the stimulus was present (by pressing “j” for *yes*) or not (by pressing “n” for *no*). If participants were not able to decide within 3500 ms, the stimulus disappeared and they were urged to decide immediately. Upon making a decision, the participants were further asked to indicate their level of certainty by pressing number keys 1 (*absolutely uncertain*) to 5 (*absolutely certain*) on their keyboard. Participants completed two experiment blocks consisting of 100 trials each. In the first block, 50% of the trials contained a signal. In the second block, participants were randomly assigned to either a “low base rate” (25% signal stimuli) or the “high base rate” (75% signal stimuli) condition. Participants were informed about the base rate change and that they could expect one in four (low base rate condition) or three in four (high base rate condition) images to contain the stimulus, respectively. During the whole experiment, stimulus difficulty was held constant among participants as each participant was presented with the same amount of images of each difficulty. Stimuli were presented in random order, and each image was presented only once. After completing both experiment blocks, irrational beliefs were assessed.

### Analysis

#### Data preprocessing

From the collected responses, only valid and complete observations were selected (N = 144,600 trial responses from 723 participants). For each participant, experiment trials of block 1 and 2 were sorted into one of four categories: hit (signal present, response *yes*), miss (signal present, response *no*), false alarm (signal absent, response *yes*) and correct rejection (signal absent, response *no*). Based on this classification, the perceptual sensitivity index ($d^{\prime}$, see Eq. 1) as well as response bias (*c*, see Eq. 2) were calculated for each participant, image category and base rate level applying the *dprime* function from the psycho package in R^46^. We further calculated $\Delta$*c* as a measure of individual criterion shifting by calculating the difference in response criterion *c* between block 1 and block 2 trials.

1 $$\begin{aligned} d^{\prime }=Z\left( P_{h i t}\right) -Z\left( P_{F A}\right) \end{aligned}$$

$d^{\prime }$: perceptual sensitivity; $Z\left( P_{h i t}\right)$: z-value associated with the probability of a hit; $Z\left( P_{F A}\right)$: z-value associated with the probability of a false alarm

2 $$\begin{aligned} c=-\frac{Z\left( P_{h i t}\right) +Z\left( P_{F A}\right) }{2} \end{aligned}$$

*c*: response criterion (bias); $Z\left( P_{h i t}\right)$: z-value associated with the probability of a hit; $Z\left( P_{F A}\right)$: z-value associated with the probability of a false alarm.

To assess whether individual images were disproportionately susceptible to misclassifications, Rosner outlier test was conducted on mean correct classifications of individual stimuli. No outliers were identified for signal stimuli, for noise images however two individual images were indicated by the Rosner outlier test which were disproportionately classified as ’signal’ by participants. Upon visual inspection of the corresponding image files, the removal of trials containing these stimuli (n = 1130 trials) was deemed justified as these two noise images indeed seemingly contain accidental face features (see Fig. S1 in the Supplemental Materials associated with this article).

The aforementioned preprocessing steps resulted in an analysis data set consisting of N = 143458 signal detection trials performed by N = 723 participants.

#### Preliminary analysis and descriptive results

We first report the descriptive statistics and intercorrelations of the three belief variables, and also the descriptive statistics of the dependent variables of interest (false alarms, sensitivity, response bias). We also explored how these variables were related to age, sex, and the educational level. Specifically, for the three demographic variable, the zero-order correlation (Spearman’s rho) with each of the belief variable was computed. With regards to false alarms, sensitivity and response bias, each of the three demographic variable was entered into the respective baseline model (described below) and tested against the baseline model (without the demographic variable) via likelihood ratio test. For the analysis of age, two participants with missing values were excluded. For the analysis of gender, participants who indicated “other” (*n* = 20) were excluded. Finally, for the analysis of the educational level, the lowest (4.6%) and second lowest (27.4%) educational levels were merged, and the variable was treated as continuous predictor.

#### Main analysis

The main analysis focuses on those dependent variables that allow for a direct test of our hypothesis. First, false alarm rate (FAR) reflects the amount of illusory pattern perception. Second, sensitivity ($d^{\prime }$) reflects the ability to distinguish signal from noise. And finally, the response bias (*c*) reflects the proneness of a participant to respond *yes* (or *no*, respectively). In a first step, a baseline model was computed for each dependent variable (false alarm rate, sensitivity, response bias) with the predictors image category (face, house), base rate (25%, 50% and 75%), and the interaction between image category and base rate. Specifically, false alarms were analysed by a generalized linear mixed effects logistic regression at the trial level (1 = *yes*-response, 0 = *no*-response) for the 100% noise trials, with image category, base rate and the interaction as fixed effect predictor, and participant and stimulus as random slopes. Sensitivity $d^{\prime}$ and response bias *c* was analysed by a linear mixed effect model with image category, base rate, and the interaction as fixed effect predictor, and participant as random intercept effect. The effects of the task-manipulations (image category and base rate) are reported first. In a second step, the effect of the belief variables was assessed by entering each belief variable separately in each of the three baseline models and by testing the model with the belief variable against the model without the belief variable (i.e., the baseline model) via likelihood-ratio tests. This approach reveals whether there is an overall effect of the belief variables, taking into account the different task conditions and the repeated measurement structure (since all participants completed two blocks, one with the 50%, and one with either 25% or 75% signal base rate). In a third step, the interaction between the belief variable and image category, or respectively the interaction between the belief variable and base rate was entered separately as additional fixed effect predictor into the models, and tested against the respective model without this additional interaction term. This allows to test possible moderating effects of image category or base rate for the effect of irrational belief endorsement on false alarms, sensitivity or response bias. Analyses were computed in R using the lme4 package^47^. Because the belief variables differed with respect to scale range (1-7 vs. 1-11), belief variables were z-transformed for comparability. The estimates of the belief variables therefore reflect the increase in false alarm rate, sensitivity or response bias when the belief variable is increased by one standard deviation. The estimates can be interpreted as effect sizes.

## Results

### Descriptive statistics

#### Belief variables

Descriptive statistics of the belief variables, their intercorrelation, and their correlations with age and sex is summarized in Table 1. As expected, the three belief variables were highly intercorrelated. Moreover, paranormal belief was significantly higher in female than in male participants (female: *M* = 3.73, *SEM* = 0.06; male: *M* = 2.85, *SEM* = 0.07; *p* <. 001). The same was true for conspiracy mentality (female: *M* = 3.29, *SEM* = 0.06; male: *M* = 3.00, *SEM* = 0.06) and for COVID-19 conspiracy beliefs (female: *M* = 3.49, *SEM* = 0.11; male: *M* = 3.00, *SEM* = 0.12). Furthermore, education level was negatively associated with paranormal beliefs ($\textit{r}_{\textit{s}}$ = -.22), with conspiracy mentality ($\textit{r}_{\textit{s}}$ = -.29), and also with COVID-19 conspiracy beliefs (($\textit{r}_{\textit{s}}$ = -.31, all *p*s < .001).

**Table 1:** Descriptive statistics and correlations of irrational belief variables.

	Descriptive statistics			Correlations			
	*M (SD)*	Skew	$\alpha$	Age	Gender	Conspiracy mentality	COVID-19 conspiracy
Paranormal beliefs	3.33 (1.30)	0.24	.91	.04	.33***	.55***	.47***
Conspiracy mentality	3.15 (1.11)	0.29	.84	.01	.14***	–	.66***
COVID-19 conspiracy	3.27 (2.17)	1.21	.93	-.01	.12**	–	–

*N* = 723. $\alpha$ = Cronbach’s alpha. For the correlations with age, two participants with missing values were excluded. For the correlations with gender, ten participants who indicated other than male or female were excluded; female = 1, male = 0. **p < .01; ***p < .001.

#### Dependent variables

The mean false alarm rate (FAR) was *M* = 0.26 (*SEM* = 0.01), mean sensitivity $d^{\prime }$ was *M* = 1.57 (*SEM* = 0.01), and the mean response bias *c* was *M* = $-0.02$ (*SEM* = $-0.01$). False alarm rate was right skewed (skew = 0.9), and visual inspection of distribution and Q-Q-plots (see Fig. S2 in the Supplemental Materials) confirm non-normality of false alarm rates. This confirms the appropriateness of our approach of analyzing false alarms as binary logistic regression at the trial level instead of at the aggregated FAR level. Sensitivity (skew = 0.18) and response bias (0.05) were normally distributed, as confirmed by visual inspection of distribution and Q-Q-plots (see Fig. S2 in the Supplemental Materials). False alarm rate, sensitivity, and response bias were neither influenced by age (all *p*s > .462), nor by sex (all *p*s > .480) or by education level (all *p*s > .174). Demographic variables were therefore not further considered in the main analyses.

### Effects of image category and base rate on task performance (baseline models)

#### False alarm rate

For the false alarm rate, model comparison for the three fixed effects image category, base rate and the interaction revealed that all three effects were significant; image category: $\chi ^2$ (1) = 52.27, *p* < .001, base rate: $\chi ^2$ (2) = 519.67, *p* < .001, interaction: $\chi ^2$ (2) = 226.90, *p* < .001. The false alarm rate was higher for house stimuli (*M* = 0.25, *SEM* = 0.01) than for face stimuli (*M* = 0.17, *SEM* = 0.01). Moreover, false alarm rate increased with increasing base rate (25%: *M* = 0.15, *SEM* = 0.01, 50%: *M* = 0.22, *SEM* = 0.01, and 75%: *M* = 0.26, *SEM* = 0.01).

#### Sensitivity

Model comparison for the three fixed effects image category, base rate and the interaction revealed that all three effects were significant; image category: $\chi ^2$ (1) = 169.02, *p* < .001, base rate: $\chi ^2$ (2) = 21.45, *p* < .001, interaction: $\chi ^2$ (2) = 13.11, *p* = .001. Sensitivity was higher for face stimuli (*M* = 1.76, *SEM* = 0.02) than for house stimuli (*M* = 1.39, *SEM* = 0.02). Sensitivity was independent of the base rate for face stimuli (*p*s > .801 for all pairwise comparisons). In contrast, sensitivity was influenced by the base rate for house stimuli, with a higher value for the 25% base rate condition when compared to the 50% (*p* < .001) or 75% base rate condition (*p* < .001).

#### Response bias

The overall response bias was $-0.01$ (*SEM* = 0.02). Model comparison for the three fixed effects image category, base rate and the interaction revealed that all three effects were significant; image category: $\chi ^2$ (1) = 6.07, *p* = .014, base rate: $\chi ^2$ (2) = 110.29, *p* < .001, interaction: $\chi ^2$ (2) = 44.01, *p* < .001. There was a conservative response bias for face stimuli (*M* = 0.03, *SEM* = 0.02) and a liberal response bias for house stimuli (*M* = $-0.05$, *SEM* = 0.02). Moreover, the response bias was conservative for the 25% base-rate (*M* = 0.15, *SEM* = 0.02), liberal for the 50% base rate (*M* = $-0.05$, *SEM* = 0.02) and most liberal for the 75% base-rate (*M* = $-0.13$, *SEM* = 0.02). All pairwise comparisons between the three levels of base rate are significant (*p*s < .001).

**Figure 2:**
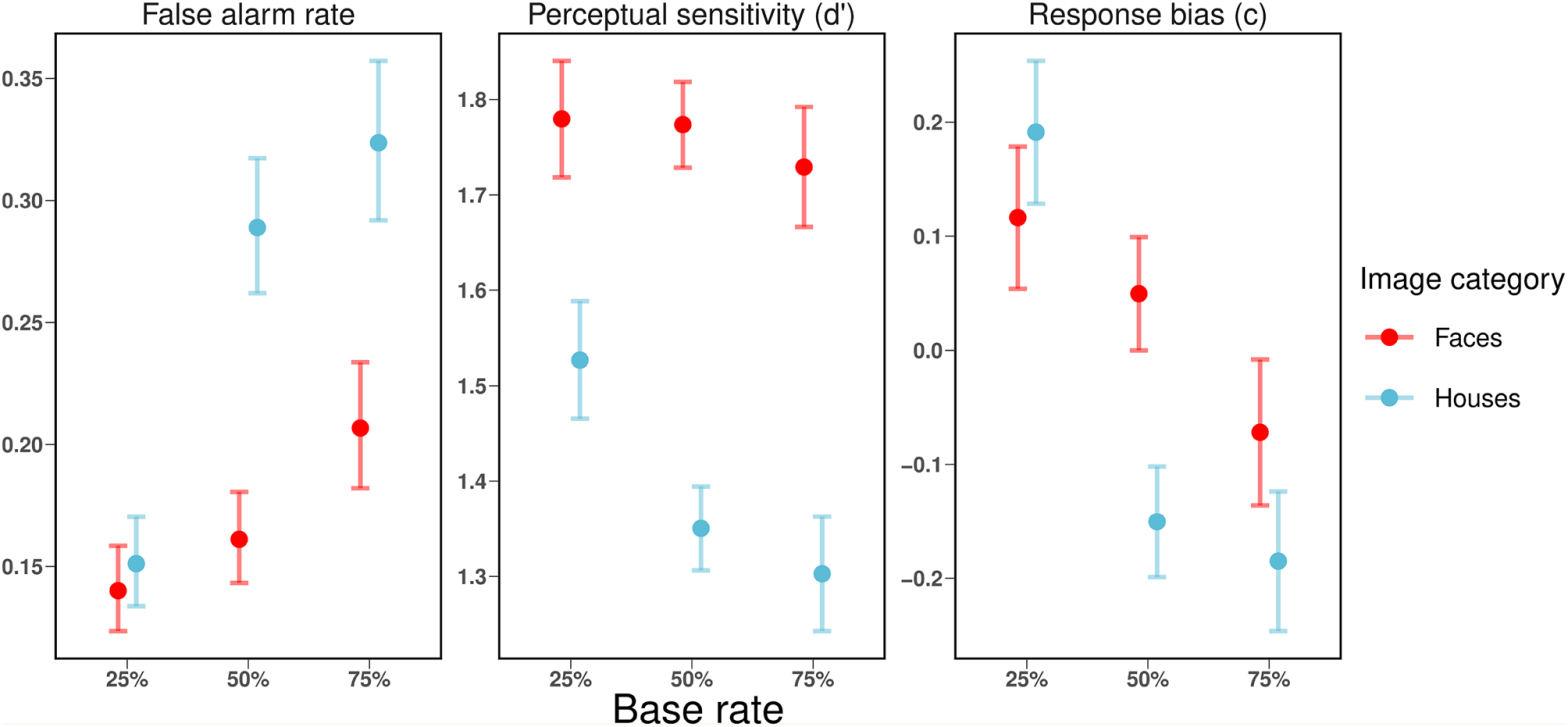
Interactions with image category and base rate: Means of the three dependent variables false alarm rate, perceptual sensitivity ($d^{\prime }$) and response bias (*c*) with respect to image category and base rate condition. The error bars represent the Standard Error of the Mean (*SEM*).

For an overview of these effects and interactions see Fig. 2.

### Associations with irrational beliefs

**Figure 3:**
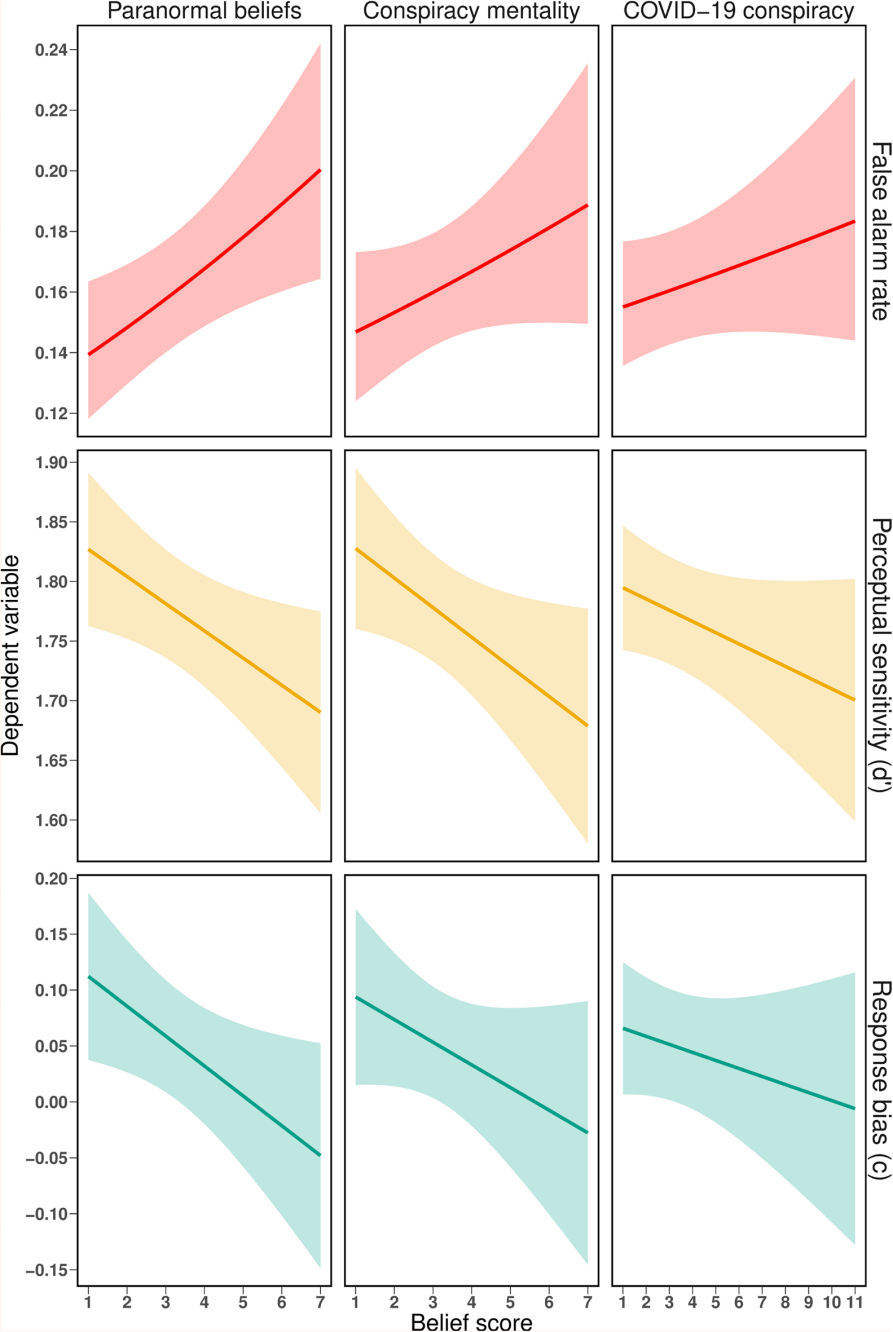
Associations between dependent variables and irrational beliefs: *False alarm rate* was positively associated with all three irrational beliefs, but only was significant for paranormal beliefs. *Perceptual sensitivity* was negatively associated with all three irrational beliefs, but this association was not significant for COVID-19 conspiracy beliefs. *Response bias* was negatively associated with all three irrational beliefs, but this association was only significant for paranormal beliefs.

For an overview of the associations between dependent variables and irrational beliefs, see Fig. 3.

#### False alarm rate

**Figure 4:**
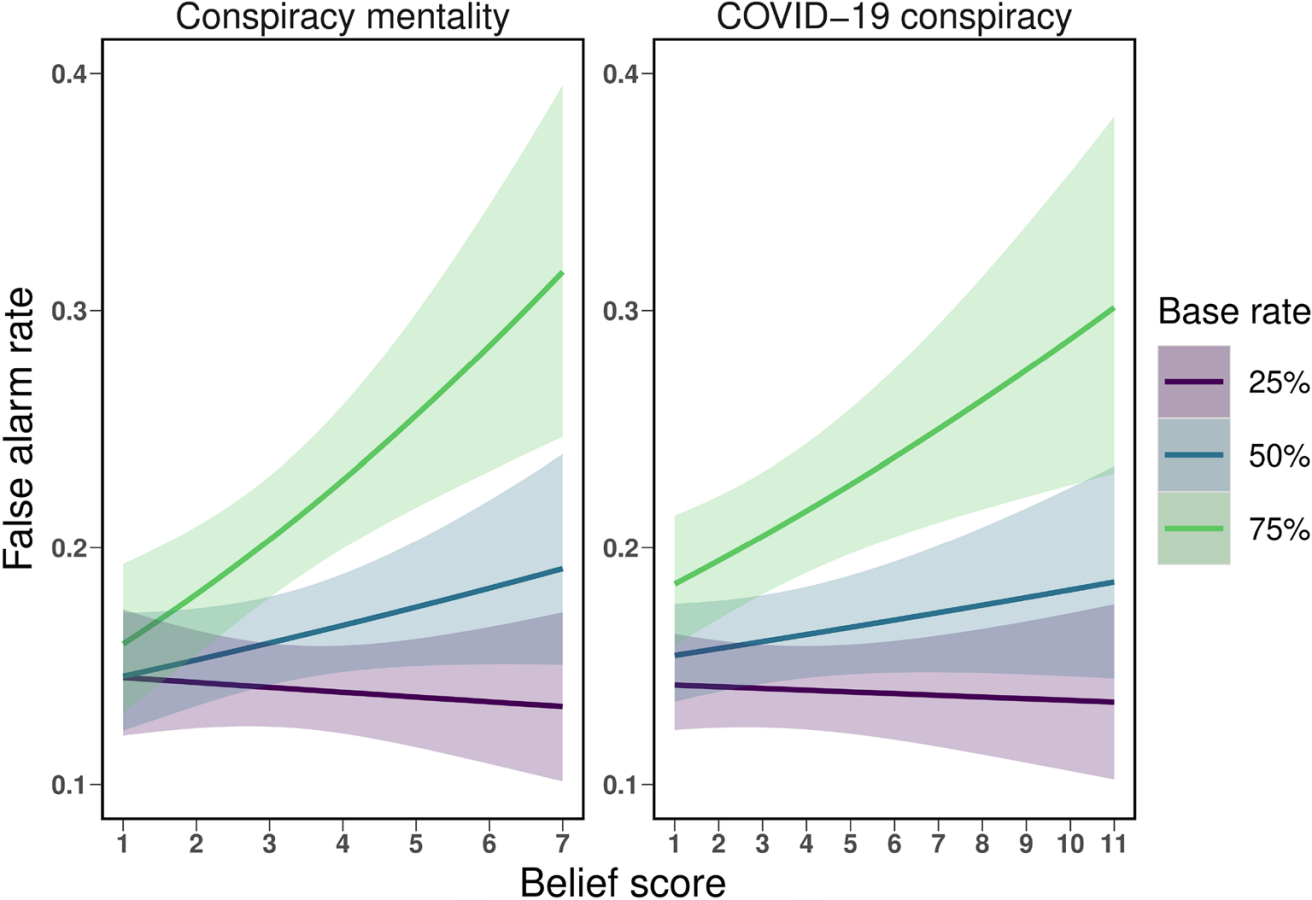
Interactions between irrational beliefs (conspiracy mentality, COVID-19 conspiracy beliefs) and base rate for false alarm rates.

All three irrational belief variables were positively associated with the false alarm rate (paranormal beliefs: estimate = 0.094, *SEM* = 0.036; conspiracy mentality: estimate = 0.056, *SEM* = 0.036; COVID-19 conspiracy beliefs: estimate = 0.044, *SEM* = 0.037), but only paranormal belief reached significance (see Table 2). Specifically, an increase in one *SD* in the proneness to the paranormal increased the probability of making a false alarm by 9.43%. Interestingly, for conspiracy mentality and COVID-19 conspiracy beliefs, there was a significant interaction between belief and base rate (see Table 2). Thus, the relationship between conspiracy beliefs and false alarm was moderated by base rate, as illustrated in Fig. 4. Follow-up analyses show that the positive association was only present for the 75% base rate condition (conspiracy mentality: estimate = 0.149, *SEM* = 0.041, *p* < .001; COVID-19 conspiracy beliefs: estimate = 0.064, *SEM* = 0.021, *p* = .002, but not for the 25% or 50% base rate conditions (*p*s >. 05).

**Table 2:** Summary of model comparisons for irrational beliefs and dependent variables.

Effect	Paranormal beliefs		Conspiracy mentality		COVID-19 conspiracy	
	$\chi ^{2}$	*p*	$\chi ^{2}$	*p*	$\chi ^{2}$	*p*
False alarm rate						
Belief (B)	6.79	.009	2.36	.125	1.45	.228
B $\times$ Image category	0.29	.593	0.98	.321	0.88	.349
B $\times$ Base rate	2.15	.341	21.82	< .001	14.90	< .001
Perceptual sensitivity ($d^{\prime}$)						
Belief (B)	5.19	.023	4.50	.034	2.47	.116
B $\times$ Image category	0.98	.323	0.34	.559	0.08	.782
B $\times$ Base-rate	2.75	.252	4.88	.087	4.96	.084
Response bias (*c*)						
Belief (B)	4.79	.029	2.01	.156	0.96	.327
B $\times$ Image category	0.08	.782	1.24	.265	1.47	.226
B $\times$ Base-rate	0.04	.978	3.83	.148	1.15	.562

#### Sensitivity

Paranormal beliefs (estimate = $-0.030$, *SEM* = 0.013) and conspiracy mentality (estimate = $-0.028$, *SEM* = 0.013) were negatively associated with sensitivity. COVID-19 conspiracy belief was not associated with sensitivity (estimate = $-0.020$, *SEM* = 0.013), and there were no moderating effects of stimulus category or base rate (see Table 2).

#### Response bias

Paranormal belief was negatively associated with the response bias (estimate = $-0.035$, *SEM* = 0.016), thus with a more liberal response behavior. Neither conspiracy mentality (estimate = $-0.023$, *SEM* = 0.016) nor COVID-19 conspiracy beliefs (estimate = $-0.016$, *SEM* = 0.016) were significantly associated with response bias, and there were no moderating effects of image category or base rate (see Table 2). None of the three belief variables was correlated with the extent of response criterion shift $\Delta$*c* from the first block (50% signals) to the second block (25% or 75% signals) (all *p*s > 0.162) see Table S1 in the Supplemental Materials for an overview).

### Additional analysis

#### Miss rate

Since we found that irrational beliefs (at least paranormal beliefs) are associated with both a higher false alarm rate and a more liberal response criterion, we explored whether the more liberal response criterion reflects simply a general yes-say tendency. If the latter is the case, then the miss rate should be negatively associated with irrational belief endorsement, as more *yes* responses lead to fewer misses. To explicitly test for this, we repeated the analysis for the miss rate. In analogy to the analysis above, we first computed baseline models for the miss rate, and then compared these baseline models to the models that include the belief variables. Most importantly, the analysis showed that none of the belief variables was associated with the miss rate (e.g., paranormal beliefs: *p* = .265). This indicates that the association between irrational beliefs and a more liberal response criterion can be explained by increased false alarm rates rather than by a general yes-say tendency. The full report of this analysis can be found in the Supplemental Materials associated with this article, page 5.

#### Confidence and response times

Since irrational beliefs seem to be particularly associated with a different behavior for pure noise trials, we further explored to what extent this behavior is also reflected in the confidence ratings and response times. Regarding confidence, there was a significant positive association between all three belief variables and response confidence for false alarms (all *p*s < .003). In contrast, there was no significant association between response confidence in signal trials and paranormal beliefs (*p* = .169) and conspiracy mentality (*p* = .113). The association between response confidence in signal trials and COVID-19 conspiracy beliefs was significant (*p* = .019). Thus, at least for paranormal beliefs and conspiracy mentality, greater belief endorsement does not seem to be associated with higher confidence in general but specifically with a higher confidence when perceiving illusory patterns. Regarding response times, there was no significant association between paranormal beliefs and response time for false alarms (*p* = .232). However, there was a significant negative association for conspiracy mentality (*p* = .020) and COVID-19 conspiracy beliefs (*p* = .007). In contrast, none of the belief variables was associated with response time for signal trials (all *p*s > .097). Thus, irrational beliefs were not associated with faster responses for perceptual decisions in general but—at least for conspiracy beliefs—with faster responses when detecting illusory patterns. The full report of the analysis of confidence and response time can be found in the Supplemental Materials associated with this article, pages 5–7.

## Discussion

The aim of this study was to assess the association between different types of irrational beliefs and illusory perception of visual patterns. Despite the interrelationship among the three belief variables, their association with each of these parameters varied considerably, and the application of signal detection theory allowed for a more nuanced interpretation.

In line with our expectations and previous studies^30,31^, greater paranormal belief endorsement was found to be associated with an increased tendency to perceive patterns when there are none (false alarm rate), a more liberal response criterion and a decreased perceptual sensitivity—independent of image category or base rate. Furthermore, paranormal beliefs were not associated with a diminished miss rate—which one might expect in the case of a generally increased yes-say tendency—suggesting that this effect is selective for the perception of illusory patterns, and that the more liberal response criterion stems from the increased tendency to produce false alarms.

For conspiracy beliefs, a similar pattern emerged but it did not reach significance (see Fig. 3). Instead, we found that the association between belief and false alarm rate for both conspiracy mentality and COVID-19 conspiracy beliefs was moderated by base rate. When the chance of a true meaningful pattern occurring was high, the relationship between conspiracy beliefs and false alarm rate was most prevalent, while this association disappears under equal or low base rate circumstances and is insignificant overall. This finding may be explained by several, potentially additive factors: First, a high base rate condition suggest that the absence of a signal is unlikely—further facilitating illusory pattern perception. Particularly, people with high conspiracy mentality might be more biased by the many experiences of detecting a pattern from preceding signal trials and consequently make more false alarms. Second, the tendency to produce more false alarms might be amplified by the high base rate condition, because in this condition, the absolute number of noise trials was low, and consequently even few false alarms can considerably increase the overall false alarm rate. Conspiracy mentality was also associated with a decreased ability to distinguish signal from noise, but not necessarily due to a biased response. Finally, COVID-19 conspiracy belief was neither significantly related to sensitivity nor to response bias, suggesting that, at least under the conditions present in this study, this specific form of irrational beliefs is not associated with these more general parameters of perceptual decision making.

Our findings suggest that the association between this form of illusory pattern perception and paranormal beliefs may be stronger compared to conspiracy theories. One possible explanation for this is that visual perception may play a more prominent role in the domain of paranormal beliefs, as paranormal beliefs are more strongly associated with the sensory perception of forces, energies or entities (e.g., seeing “ghosts”) whereby conspiracy beliefs relate to perceiving more abstract connections between events in the political or societal sphere.

We further hypothesized that participant’s response criterion depends on the given signal-to-noise ratio (base rate) and shifts in accordance with changes in base rates, and we explored if this shift is less pronounced the higher the level of irrational belief endorsement. Overall, the results indicate that participants shifted their criterion in alignment with the base rate manipulation. These observations are in line with commonly observed findings from signal detection theory paradigms^35,36^. This extent of this criterion shifting however was not associated with irrational beliefs, meaning that beliefs had no influence whatsoever on whether or not participants were able to integrate base rate information into their decision making process.

The additional analysis of confidence ratings and response times further underpins the aforementioned association between irrational beliefs and illusory pattern perception, specifically. The results indicate that paranormal beliefs and conspiracy mentality were associated with higher confidence when answering *yes* when no signal is present but not with a higher confidence when making judgements when signals are present. Similarly, high conspiracy belief (mentality and COVID-19) was associated with faster response times specifically for false alarms but not for signal trials. This shows that the associations found in this study cannot be attributed to global difference in speed-accuracy trade-offs. Rather, the confidence and response time patterns might reflect a higher reliance on intuitive information processing^48^. Specifically, people with higher conspiracy beliefs might rely more on their automatic, intuitive pattern detector when confronted with ambiguous information, which underlies both the development of conspiracy beliefs, and also the perception of illusory patterns in the present study^28,49^.

Overall, the observed effects of irrational beliefs on pattern perception were small, especially when compared to the other sources of variance such as image category or base rate. It needs to be further established if and how this behaviour can be generalized to the endorsement of irrational explanations in the context of real world events. If we can also observe an increased tendency to perceive illusory connections (false alarms), but not an increased tendency to dismiss weak but real explanations (misses) in people susceptible to irrational beliefs confronted with uncertainty, we might be able to derive valuable advice for policy makers, scientists and public entities who need to communicate and explain real world events.

Image category impacted people’s ability to discriminate between noise and signal, despite the attempt to create stimuli of comparable difficulty. Participants in general were more sensitive to face stimuli, even though face stimuli contained more noise. This might very well be due to a human proneness to face perception^38^, but there might be other image set characteristics that could explain this difference in sensitivity: Face stimuli were relatively uniform with not much variation in the presented faces while there was considerably more variety in house images. This might explain why participants in the houses condition had a more liberal criterion compared to the faces condition. A more varied stimulus set might elicit more creative responses and might therefore set the circumstances under which a relationship between irrational beliefs and illusory pattern perception can be more clearly observed^29,50^. Future studies should take stimulus set characteristics into account. Furthermore, base rate moderated the relationship between conspiracy beliefs and false alarm rate, further evoking questions regarding the impact of base rate information in the context of conspiracy thinking. It has to be noted that the sample in the present study, albeit large, was non-representative and the general endorsement of irrational beliefs was rather low, especially for COVID-19 conspiracy beliefs (see Fig. S3 in the Supplemental Materials), and that he testing environment was not standardized as participants completed the study online in various locations rather than in a controlled laboratory setting.

To conclude, we found paranormal beliefs to be associated with a diminished ability to distinguish signal from noise, resulting from a tendency to produce more false alarms in particular. We further conclude that how a person performs in an illusory pattern detection task might depend much more on the specific task setting than on individual belief systems, and interactions thereof can not be ruled out. Our results imply that such task characteristics need to be carefully considered and taken into account when assessing individual differences in illusory pattern perception.

## Supplementary Information

Supplementary Information.

## Data Availability

The study reported in this article was not preregistered. Deidentified data for this experiments along with the data analysis scripts as well as stimulus materials used in this study are publicly accessible at https://osf.io/rmx5z/?view_only=5b94cec092cf4d1eb7f0e728aed56826. The complete questionnaires used in this study are included in the Supplemental Material associated with this article. We confirm that we have reported all measures, conditions, data exclusions and information regarding the sample size used in this study.
