# Supplementary material for: Linking paranormal and conspiracy beliefs to illusory pattern perception through signal detection theory
Source: Sci Rep. 2023 Jun 16;13:9739. doi: 10.1038/s41598-023-36230-0 (PMC10275861; doi:10.1038/s41598-023-36230-0)
Supplement: Supplementary file 1 — Supplementary Information. [file 41598_2023_36230_MOESM1_ESM.pdf]

Supplemental materials - Linking Paranormal and Conspiracy Beliefs to Illusory Pattern Perception through Signal Detection Theory

Petra Müller[1,2,*] **and Matthias Hartmann**[1,2]

[1]UniDistance Suisse, Faculty of Psychology, Brig, 3900, Switzerland
[2]Universität Bern, Institute of Psychology, Bern, 3012, Switzerland
[*]petra.mueller@unibe.ch

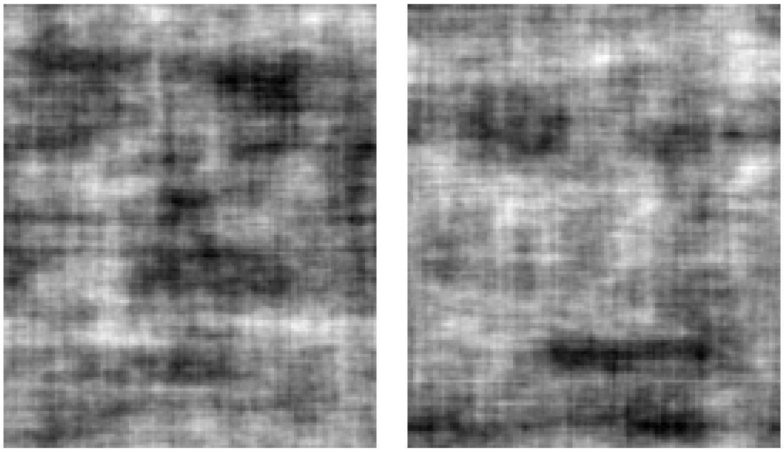

Figure S1. Noise stimuli containing accidental faces.

Condition		Association with Δc Pearson's r	p
Low base rate	Paranormal beliefs	$r(722) = .005$	.891
	Conspiracy mentality	$r(722) = .047$	.201
	COVID-19 conspiracy	$r(722) = .035$	.340
High base rate	Paranormal beliefs	$r(722) = .000$	.994
	Conspiracy mentality	$r(722) = -.052$	.163
	COVID-19 conspiracy	$r(722) = -.029$	.440

Table S1. Correlations between response criterion shift and irrational beliefs

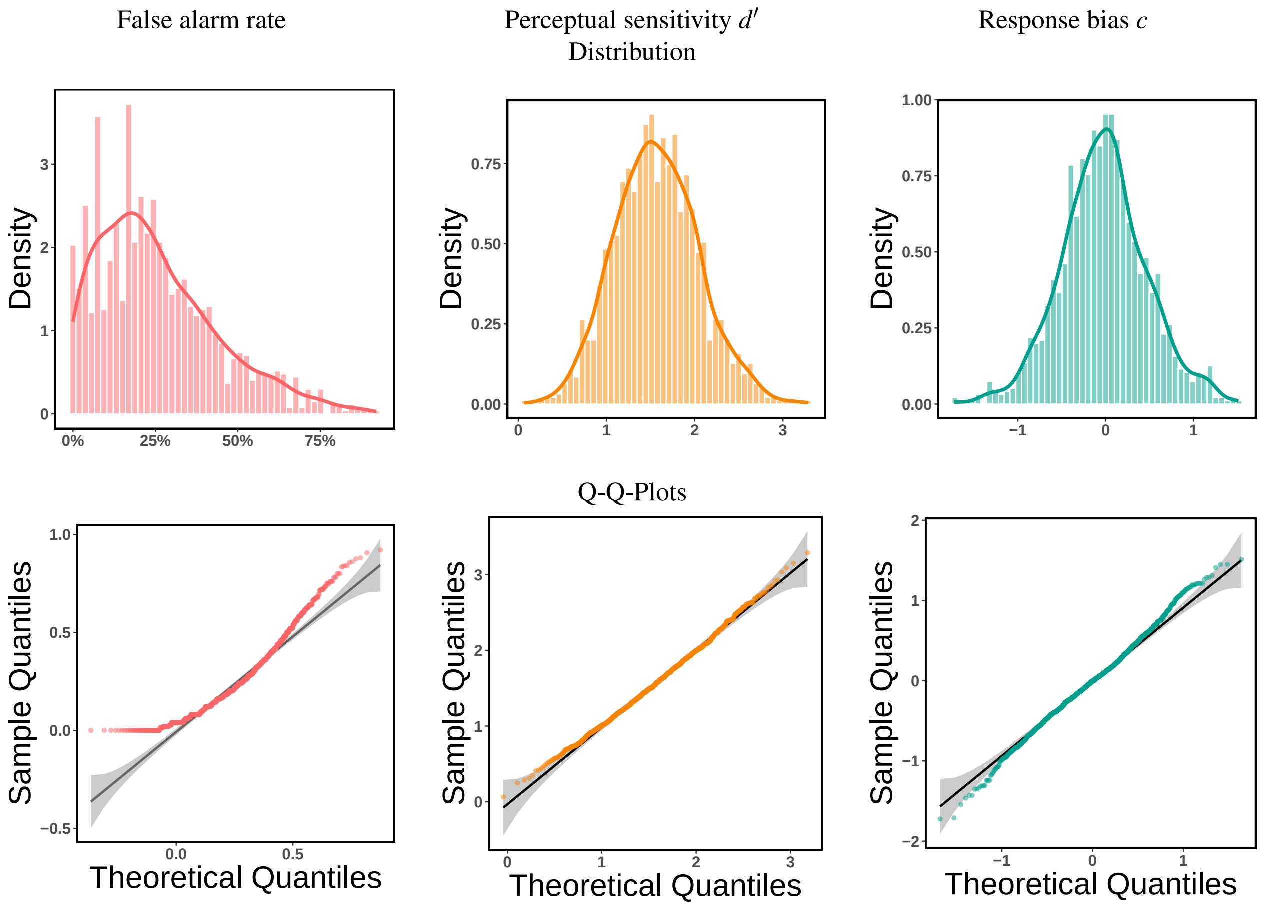

Figure S2. Density distribution and Q-Q-plots of dependent variables. False alarm rate is defined as the number of false alarms out of all noise trials.

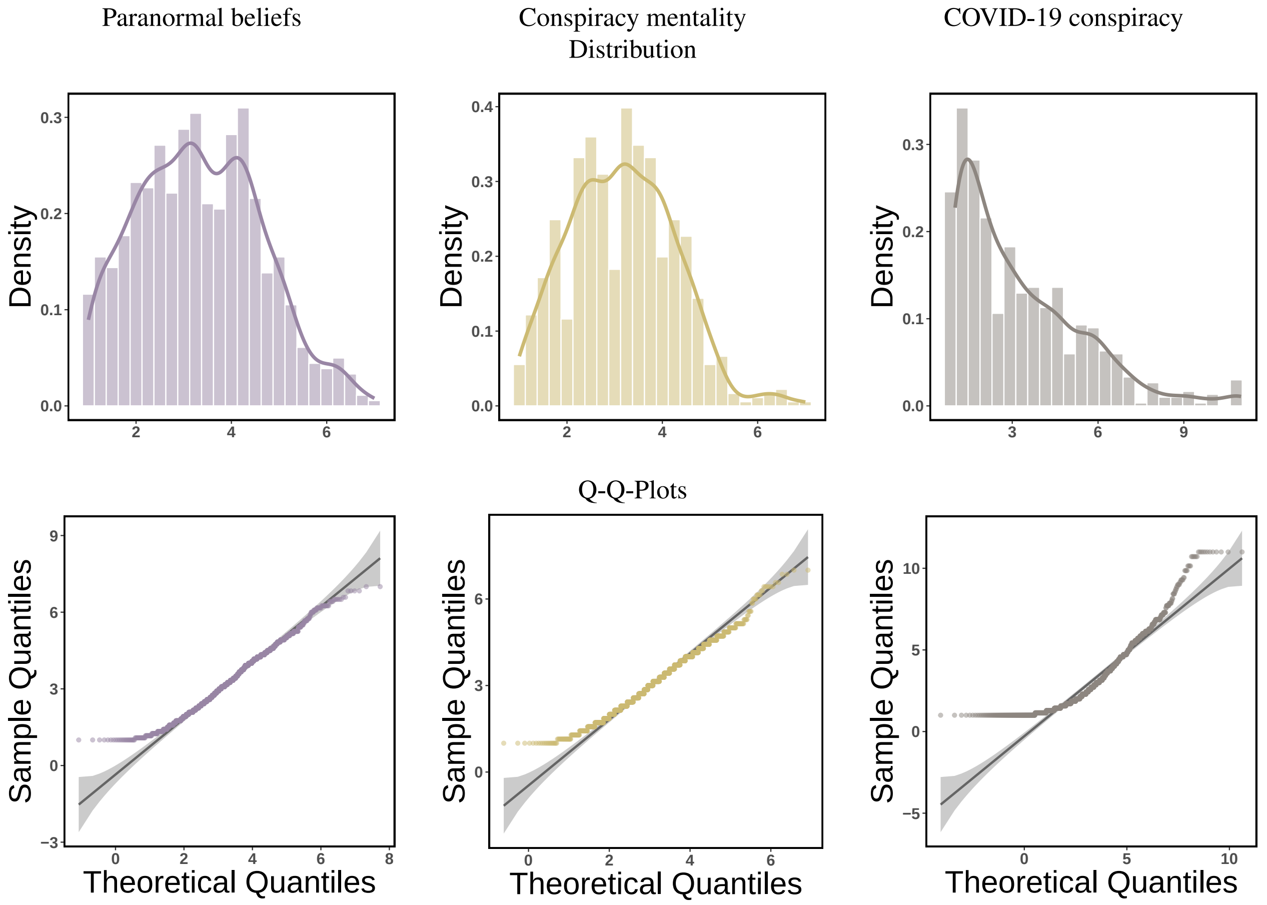

Figure S3. Density distribution and Q-Q-plots of irrational belief variables. Shown is the individual scale mean before z-transformation.

English	German
Sometimes I feel it when someone is thinking about me.	Manchmal spüre ich es, wenn jemand an mich denkt.
Events can be influenced by the power of thoughts in a way that is not yet explainable.	Geschehnisse können durch die Kraft der Gedanken auf bisher unerklärbare Weise beeinflusst werden.
My intuition sometimes tells me that certain events or objects have a special meaning, even if there is no explanation for it.	Meine Intuition sagt mir manchmal, dass gewisse Ereignisse oder Gegenstände eine spezielle Bedeutung haben, auch wenn es dafür keine Erklärung gibt.
Some decisions or events in our lives are influenced by experiences we have had in a previous life.	Manche Entscheidungen oder Geschehnisse in unserem leben werden von Erfahrungen beeinflusst, die wir in einem früheren Leben gemacht haben.
There are good and evil forces that influence our lives (e.g. divine beings, spirits, guardian angels).	Es gibt gute und böse Kräfte, welche unser Leben beeinflussen (z.B. göttliche Wesen, Geister, Schutzengel).
The full moon has a not yet explainable effect on people's minds.	Der Vollmond hat eine bisher unerklärte Wirkung auf die Psyche.
There are ways of predicting the future or obtaining hidden information that go beyond previously explainable approaches (e.g. by means of horoscopes, card reading, epiphany, pendulum).	Es gibt Möglichkeiten, die Zukunft vorherzusagen oder an verdeckte Informationen zu gelangen, die über bisher erklärbare Zugänge hinausgehen (z.B. mittels Horoskope, Kartenlegen, Eingebungen, Pendeln).
Sometimes I notice unusual events or signs that nobody else notices.	Manchmal fallen mir ungewöhnliche Ereignisse oder Zeichen auf, die sonst niemandem auffallen.
Some people have an extrasensory ability to read the thoughts of others or transfer them to others.	Manche Menschen haben eine übersinnliche Fähigkeit, Gedenken von anderen zu lesen oder auf andere zu übertragen.
I like to engage in esoteric or spiritual topics.	Ich beschäftige mich gerne mit esoterischen oder spirituellen Themen.
Sometimes I have the feeling of receiving or losing energy when certain people look at me or touch me.	Manchmal habe ich das Gefühl, Energie zu empfangen oder zu verlieren, wenn bestimmte Menschen mich anschauen oder berühren.
Certain items (e.g. amulets, stones) or rituals bring good luck.	Bestimmte Gegenstände (z.B. Amulette, Steine) oder Rituale bringen Glück.

Table S2. 12 items of Proneness to the Paranormal Scale[1]. The items were rated on a 7 point scale ranging from 1 (*I do not agree at all*) to 7 (*I fully agree*).

English	German
The government or covert organizations are responsible for events that are unusual or unexplained.	Für ungewöhnliche oder unerklärliche Ereignisse ist die Regierung oder geheime Organisationen verantwortlich.
The alternative explanations for important societal events are closer to the truth than the official story.	Alternative Erklärungen für wichtige gesellschaftliche Ereignisse liegen näher an der Wahrheit als die offizielle Darstellung.
Many so called "coincidences" are in fact clues as to how things really happened.	Viele sogenannte "Zufälle" liefern in Tat und Wahrheit Hinweise darauf, was wirklich passiert ist.
Events throughout history are carefully planned and orchestrated by individuals for their own betterment.	Ereignisse im Laufe der Geschichte werden von Personen sorgfältig geplant und zu ihrem eigenen Vorteil inszeniert.
Many situations or events can be explained by illegal or harmful acts by the government or other powerful people.	Viele Ereignisse oder Situationen lassen sich durch illegale oder schädliche Handlungen der Regierung oder anderer mächtiger Personen erklären.
Some things that everyone accepts as true are in fact hoaxes created by people in power.	Manche Dinge, die von allen als wahr akzeptiert werden, sind in Wirklichkeit Täuschungen, die von den Mächtigen kreiert wurden.
Events on the news may not have actually happened.	Ereignisse in den Nachrichten sind möglicherweise nicht wirklich passiert.

Table S3. 7 Items of the Conspiracy theory ideation sub-scale of the Conspiracy Mentality scale[2]. The items were translated from English to German and were rated on a 7 point scale ranging from 1 (*I do not agree at all*) to 7 (*I fully agree*).

English	German
There exist cheap and efficient methods to treat or prevent SARS-CoV-2 which are not recognized by mainstream medicine.	Es existieren günstige, wirksame Behandlungsmethoden um COVID-19 zu heilen oder vorzubeugen, die jedoch von der Schulmedizin nicht anerkannt werden.
SARS-CoV-2 was put into circulation (or, respectively, has not been stopped) in order to reduce the overcrowded human population.	COVID-19 wurde in Umlauf gebracht (bzw. nicht aufgehalten), um die zu stark gewachsene menschliche Bevölkerung zu reduzieren.
‘SARS-CoV-2 could have been stopped right at the start, but the large companies made a business out of keeping it going.	Die COVID-19 Pandemie hätte gleich zu Beginn gestoppt werden können, aber die grossen Unternehmen machten ein Geschäft daraus, es am Laufen zu halten.
SARS-CoV-2 is not very different from an ordinary flu but is reframed as being dangerous by pharmaceutical companies to increase the sales of medication and vaccines.	COVID-19 unterscheidet sich nicht wesentlich von einer gewöhnlichen Grippe, wird jedoch von Pharmaunternehmen als gefährlich dargestellt, um den Verkauf von Medikamenten und Impfstoffen zu steigern.
The government uses the SARS-CoV-2 pandemic to justify and intensify the surveillance of citizens.	Die Regierung nutzt die COVID-19 Pandemie, um die Überwachung der Bevölkerung zu legitimieren und auszubauen.
Pharma companies use the SARS-CoV-2 pandemic to test novel vaccines and medication on a large number of people.	Die COVID-19 Pandemie wird von Pharmaunternehmen dafür ausgenutzt, neuartige Impfstoffe und oder Medikamente an einer grossen Zahl von Menschen zu testen.
The real number of SARS-CoV-2 deaths is much lower than the official numbers because most of them died because of other reasons.	Die tatsächliche Zahl von COVID-19 Verstorbenen ist viel geringer als offiziell dargestellt, weil die meisten davon aus anderen Gründen gestorben sind.

Table S4. 7 Items to assess COVID-19 conspiracy beliefs, partially adapted from Hartmann & Müller[1]. The items were rated on a 11 point scale ranging from 1 (*absolutely not*) to 11 (*certainly*).

	Paranormal beliefs		Conspiracy mentality		COVID-19 conspiracy	
Effect	χ^2	p	χ^2	p	χ^2	p
			Miss rate			
Belief (B)	1.24	.265	0.26	.613	0.01	.921
B x Image category	< 0.01	.978	1.14	.286	1.77	.183
B x Base rate	1.00	.607	0.16	.925	0.99	.610

Table S5. Summary of model comparisons for irrational beliefs and miss rate.

Additional analysis results

Analysis of miss rate

Baseline model comparison for the three fixed effects image category, base rate and the interaction revealed that the effect of base rate and the interaction was significant; image category: χ^2 (1) = 1.22, p = .270, base rate: χ^2 (2) = 133.44, p < .001, interaction: χ^2 (2) = 59.10, p < .001. The miss rate decreased with increasing base rate (25%: M = 0.10, SEM = 0.01, 50%: M = 0.07, SEM = 0.01, and 75%: M = 0.06, SEM = 0.01). The interaction shows that this decrease was more pronounced for house stimuli. Subsequent inclusion of irrational belief variables into the baseline model showed that none of the three irrational belief variables was associated with the miss rate (see Table S5).

Analysis of confidence and response times

Baseline models Confidence decreased and response times increased as a function of noise level (both effects p < .001, see Fig. S4). Furthermore, over all trials, confidence was a significant predictor of response time, estimate = -.499, SEM = 9, χ^2 (1) = 19’948, p < .001. indicating that participants responded faster when they were more confident about their decision. Furthermore, over all trials, response time was a significant predictor of accuracy, estimate = -0.0001, SEM < 0.0001, χ^2 (1) = 1847.43, p < .001. Thus, when controlling for task aspects that influence accuracy (noise level, image category, base rate), faster responses were associated with higher accuracy. This overall reversed speed-accuracy trade-off effect may be explained by the fact that responses were generally faster for easy-to-detect trials. The same association was also found when considering 100% noise trials separately, estimate < -0.0001, SEM < 0.0001, χ^2 (1) = 402.89, p < .001. This might be explained by fast

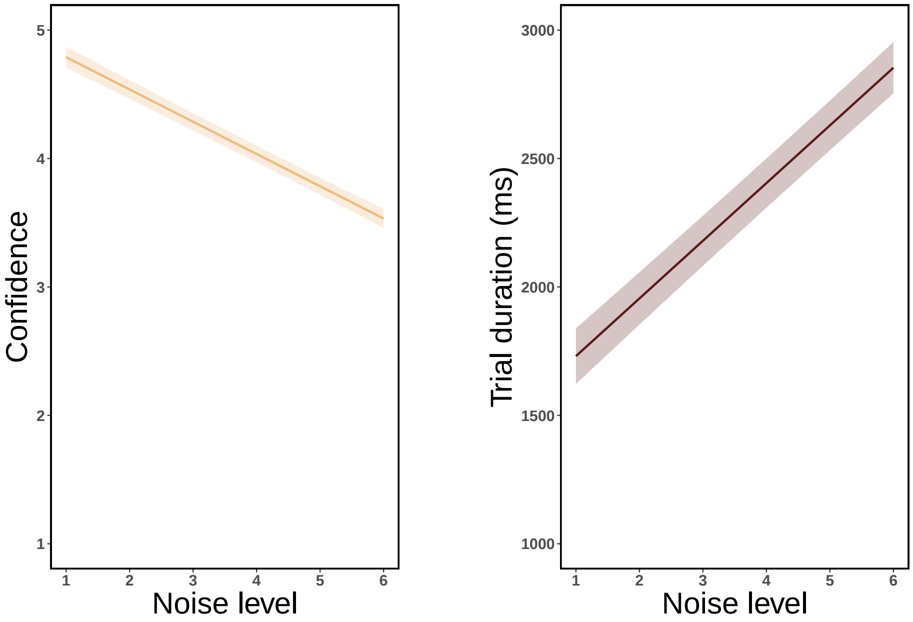

Figure S4. Confidence and response time as a function of noise level. Noise level = 6 corresponds to 100% noise stimuli.

	Paranormal beliefs		Conspiracy mentality		COVID-19 conspiracy	
Effect	χ^2	p	χ^2	p	χ^2	p
			Confidence			
False alarms	8.37	.004	8.97	.003	16.87	$< .001$
Signal trials	1.89	.169	2.51	.113	5.47	.019
			Response times			
False alarms	1.43	.232	5.43	.020	7.20	.007
Signal trials	0.27	.606	0.09	.759	2.75	.097

Table S6. Summary of model comparisons for irrational beliefs and response confidence and response time.

responses to 100% noise trials in where participants did not see any illusory pattern.

Associations with irrational beliefs To explore whether increased belief endorsement is associated with higher confidence for certain perceptual decisions, the belief variables were separately entered into the model for false alarms that included image category, base rate and their interaction as fixed effects predictor, and participant and image as random intercept effect and compared to the model without the belief variable via likelihood ratio test (analogous to the analyses reported above). There was a significant positive association between all three belief variables and confidence for false alarms (paranormal beliefs: estimate = 0.085, *SEM* = 0.029; conspiracy mentality: estimate = 0.089, *SEM* = 0.030; COVID-19 conspiracy beliefs: estimate = 0.125, *SEM* = 0.030).

To further test whether these associations are specific to the detection of illusory patterns (false alarms) or whether higher belief is associated with higher confidence when detecting patterns in general, the analysis was repeated for signal trials, including the continuous predictor noise level into the model. There was no significant association between paranormal belief and confidence for signal trials, estimate = 0.023, *SEM* = 0.017. The same held for conspiracy mentality, estimate = 0.027, *SEM* = 0.017. However, there was a significant positive association between COVID-19 conspiracy beliefs and confidence for signal trials, estimate = 0.125, *SEM* = 0.030. Thus, at least for paranormal beliefs and conspiracy mentality, higher beliefs do not seem to be associated with higher confidence in general but specifically higher confidence in false-positive judgements (see Table S6).

Paranormal belief has been related to intuitive (vs. analytical) judgment styles[3–8]. One could therefore expect that higher belief is associated with shorter response times when detecting illusory patterns. We thus repeated the analysis described above for response times. Statistical analyses are based on log-transformed response time data, but for better interpretability, estimates are in non-transformed units (ms). There was no significant association between paranormal beliefs and response time for false

alarms, estimate = -74, *SEM* = 51. In contrast, there was a significant negative association for conspiracy mentality, estimate = -108, *SEM* = 51, and also for COVID-19 conspiracy beliefs, estimate = -83, *SEM* = 52. Thus, higher conspiracy beliefs (but not paranormal belief) was associated with faster responses for false alarms. To further test whether these associations are specific for the detection of illusory patterns (false alarm) or whether they reflect general decision speed when detecting patterns, we again repeated the analysis for signal trials. There were no significant associations between the belief variables and response times for signal trials (paranormal beliefs: estimate = 30, *SEM* = 29; conspiracy mentality: estimate = 14, *SEM* = 29; COVID-19 conspiracy beliefs: estimate = -30, *SEM* = 28). Thus, higher beliefs do not seem to be associated with faster responses for pattern detection in general but specifically with faster (i.e., more intuitive) responses when detecting illusory patterns (See Table S6).

Scale	N Items
Openness subscale of the German version[9] of the NEO Personality Inventory-Revised (NEO-PI-R)[10]	48
Big 5 personality traits openness, conscientiousness, extraversion, agreeableness, and neuroticism (German short version[11])	8 (2 items per trait)
German translation[12] of the short version of the Faith in Intuition Scale[13]	3 items with the highest loadings on the intuition factor
Self-attributed need for uniqueness (SANU) scale[14]	4 (translated to German)
Paranoid Ideation Subscale of the Schizotypal Personality Questionnaire (SPQ)[15]	8 (translated to German)
Kurzskala Interpersonelles Vertrauen (KUSIV3)[16]	3
Traditional Religious Beliefs subscale of the Revised Paranormal Belief Scale (PBS)[17]	4 (translated to German)
Scepticism subscale of the Conspiracy Mentality Scale[2]	4 (translated to German)
anti-science beliefs and attitudes subscale[18]	5 (translated to German)
A self developed measurement to measure the ascription of trustworthiness, benevolence, expertise, integrity and threat to low and high power societal groups	30

Table S7. All additional scales participants were presented with. These additional scales were not related to the study at hand, and at no point during the analysis were any measurements obtained from them introduced into the analysis of illusory pattern perception. The measurements obtained from these scales are for the purpose of a different research project and the means of data collection in this study facilitates the collection of data for more than one study.

References

1. Hartmann, M. & Müller, P. Acceptance and adherence to covid-19 preventive measures are shaped predominantly by conspiracy beliefs, mistrust in science and fear – a comparison of more than 20 psychological variables. *Psychol. Reports* **0**, DOI: 10.1177/00332941211073656 (2022a). PMID: 35212558, https://doi.org/10.1177/00332941211073656.

2. Stojanov, A. & Halberstadt, J. The conspiracy mentality scale. *Soc. Psychol.* **50**, 215–232, DOI: 10.1027/1864-9335/a000381 (2019). https://doi.org/10.1027/1864-9335/a000381.

3. Swami, V., Voracek, M., Stieger, S., Tran, U. S. & Furnham, A. Analytic thinking reduces belief in conspiracy theories. *Cognition* **133**, 572–585, DOI: https://doi.org/10.1016/j.cognition.2014.08.006 (2014).

4. Stanley, M. L., Barr, N., Peters, K. & Seli, P. Analytic-thinking predicts hoax beliefs and helping behaviors in response to the covid-19 pandemic. *Think. & Reason.* **27**, 464–477, DOI: 10.1080/13546783.2020.1813806 (2021). https://doi.org/10.1080/13546783.2020.1813806.

5. Gligorić, V. *et al.* The usual suspects: How psychological motives and thinking styles predict the endorsement of well-known and covid-19 conspiracy beliefs. *Appl. Cogn. Psychol.* **35**, 1171–1181, DOI: https://doi.org/10.1002/acp.3844 (2021). https://onlinelibrary.wiley.com/doi/pdf/10.1002/acp.3844.

6. Lindeman, M. & Aarnio, K. Paranormal beliefs: their dimensionality and correlates. *Eur. J. Pers.* **20**, 585–602, DOI: 10.1002/per.608 (2006). https://doi.org/10.1002/per.608.

7. Denovan, A., Dagnall, N., Drinkwater, K., Parker, A. & Neave, N. Conspiracist beliefs, intuitive thinking, and schizotypal facets: A further evaluation. *Appl. Cogn. Psychol.* **34**, 1394–1405, DOI: https://doi.org/10.1002/acp.3716 (2020). https://onlinelibrary.wiley.com/doi/pdf/10.1002/acp.3716.

8. Pytlik, N., Soll, D. & Mehl, S. Thinking preferences and conspiracy belief: Intuitive thinking and the jumping to conclusions-bias as a basis for the belief in conspiracy theories. *Front. Psychiatry* **11**, DOI: 10.3389/fpsyt.2020.568942 (2020).

9. Ostendorf, F. & Angleitner, A. *Neo-Persönlichkeitsinventar nach Costa und McCrae: Neo-PI-R; Manual* (2004).

10. Costa, P. T. & McCrae, R. R. *Neo Pi-R* (Psychological assessment resources Odessa, FL, 1992).

11. Rammstedt, B. & John, O. Measuring personality in one minute or less: A 10-item short version of the big five inventory in english and german. *J. Res. Pers.* **41**, 203–212, DOI: 10.1016/j.jrp.2006.02.001 (2007).

12. Keller, J., Bohner, G. & Erb, H.-P. Intuitive und heuristische urteilsbildung - verschiedene prozesse? *Zeitschrift fuer Sozialpsychologie* **31**, 87–101, DOI: 10.1024//0044-3514.31.2.87 (2000).

13. Epstein, S., Pacini, R., Denes-Raj, V. & Heier, H. Individual differences in intuitive–experiential and analytical–rational thinking styles. *J. personality social psychology* **71**, 390–405, DOI: 10.1037/0022-3514.71.2.390 (1996).

14. Lynn, M. & Snyder, C. R. *Handbook of positive psychology*, chap. Uniqueness Seeking (New York, NY: Oxford University Press., 2002).

15. Raine, A. The SPQ: A Scale for the Assessment of Schizotypal Personality Based on DSM-III-R Criteria. *Schizophr. Bull.* **17**, 555–564, DOI: 10.1093/schbul/17.4.555 (1991). https://academic.oup.com/schizophreniabulletin/article-pdf/17/4/555/5352768/17-4-555.pdf.

16. Beierlein, K. C. K. A. J. . R. B., C. Interpersonales vertrauen (kusiv3). *Zusammenstellung sozialwissenschaftlicher Items und Skalen (ZIS)* DOI: 10.6102/zis37 (2014).

17. Tobacyk, J. J. A revised paranormal belief scale. *The Int. J. Transpers. Stud.* **23**, 94–98 (2004).

18. Rizeq, J., Flora, D. B. & Toplak, M. E. An examination of the underlying dimensional structure of three domains of contaminated mindware: paranormal beliefs, conspiracy beliefs, and anti-science attitudes. *Think. & Reason.* **27**, 187–211, DOI: 10.1080/13546783.2020.1759688 (2021). https://doi.org/10.1080/13546783.2020.1759688.
